# Development and Validation of Nutrition Literacy Assessment Instrument for Chinese Pregnant Women

**DOI:** 10.3390/nu14142863

**Published:** 2022-07-13

**Authors:** Yalin Zhou, Ying Lyu, Runlong Zhao, Hanxu Shi, Wanyun Ye, Zhang Wen, Rui Li, Yajun Xu

**Affiliations:** 1 Department of Nutrition and Food Hygiene, School of Public Health, Peking University, NO. 38 Xueyuan Road, Beijing 100083, China; zylyingyang@163.com (Y.Z.); lybjmu@126.com (Y.L.); 18210192169@163.com (R.Z.); 1610306136@bjmu.edu.cn (H.S.); yewanyun_vera@bjmu.edu.cn (W.Y.); 1710306240@pku.edu.cn (Z.W.); lr15321657608@163.com (R.L.); 2 PKUHSC-China Feihe Joint Research Institute of Nutrition and Healthy Lifespan Development, NO. 38 Xueyuan Road, Beijing 100083, China; 3 Beijing Key Laboratory of Toxicological Research and Risk Assessment for Food Safety, Peking University, NO. 38 Xueyuan Road, Beijing 100083, China

**Keywords:** nutrition literacy assessment instrument, pregnant women, China

## Abstract

The study was designed to develop and validate the nutrition literacy assessment instrument for pregnant women in China (NLAI-P). The dimension, components and questions of NLAI-P were identified via literature review and expert consultation. A panel of experts evaluated the content validity. The construct validity was evaluated by using the exploratory factor analyses (EFA) and confirmatory factor analyses (CFA). Cronbach’s α coefficient and split-half reliability were applied for examining the reliability. The NLAI-P was divided into 3 dimensions including knowledge, behavior and skill dimension. Findings showed NLAI-P possessed the satisfactory content validity (content validity index = 0.98, content validity ratio = 0.97), acceptable construct validity (χ^2^/*df* = 1.82, GFI = 0.86, AGFI = 0.84, RMSEA = 0.046) and good reliability (Cronbach’s α coefficient = 0.82). The average scores of NLAI-P were 46.59 ± 9.27. With the adjustment of confounding factors, education level presented a significantly positive correlation with NLAI-P scores. In conclusion, NLAI-P were valid and reliable to inspect NL level of pregnant women in China. Poor NL was prevalent among Chinese pregnant women. Based on the education level, taking targeted propaganda and education measures would achieve the optimal effect. NLAI-P can be applied as the tool for monitoring and assessing NL of pregnant women, and facilitate the designation of targeted interventions policies.

## 1. Introduction

Globally, nutrition-related chronic diseases are on the rise year by year. The role of nutrition and diet in etiology of these diseases appears more and more important. Refining dietary quality, optimizing dietary structure and altering unhealthy dietary behaviors are of practical significance for the primary prevention of nutrition-related chronic diseases [1,2,3,4]. Under the condition comes the nutrition literacy (NL). On the basis of health literacy, NL was defined as the capacity to access, process, and understand the faculty nutrition information and the skills to use the information for appropriate decision making to maintain and promote health [5]. It has been well documented that adequate NL emerges as a positive component in the prevention of nutrition-related chronic diseases including obesity, cardiovascular diseases and cancer [2,6,7]. Enhancing NL has even come as one of the strategic goals of national health policies. In 2016, the Chinese government issued the “Outline of Healthy China 2030 plan” and emphasized the improvement of people’s nutrition and health literacy [8].

Gestational period is the initial stage of early life 1000 days. The nutritional status of pregnant women does not only affect their own health, but also has short- and long-term effects on offspring health. Maternal demands for nutrition during pregnancy are much higher than normal women’s in order to maintain their own demands and fetal growth and development, which means that pregnant women are more prone to suffering from nutrition-related health problems. In China, pregnant women are still confronted with a variety of nutrition-related problems including inadequate consumption of multiple nutrients, chronic low intake of calcium and vitamin D, anemia, vitamin A and vitamin D deficiency, gestational obesity, gestational hypertension and gestational diabetes mellitus [9,10,11]. It has been documented that the lack of nutrition knowledge and healthy eating behavior as well as relevant skills may be one of the significant reasons for the occurrence of these issues during pregnancy [1]. Therefore, the improvement of the maternal NL during pregnancy acts as essential measurements to promote maternal health.

The advanced NL is characterized of the profound cognition of nutrition knowledge, optimized dietary behaviors, and highly developed skills to address nutrition and health barriers [5]. Therefore, it is taken as a “critical control point” of boosting better health outcomes of pregnant women. The evaluation of NL is the premise of improving NL, which contributes to screening the people with inadequate or poor NL and then taking targeted interventions into action. Currently, NL assessment instruments have been established and validated, such as nutrition literacy scale (NLS) for general adults in USA [12], nutrition literacy assessment instrument (NLit) for Americans [13], nutrition literacy scale for the Japanese elderly [14], food and nutrition literacy scale (FNLIT) for Iran elementary school children [7] and nutrition literacy electronic tool for Australians Adults [15]. In China, NL assessment tools for general adults [16], the elderly [17] and school-age children [18] have been developed and applied to the corresponding population in support of targeted delivery of public health services. However, there is lack of NL assessment tools for Chinese pregnant women who are susceptible to nutrition-related problems. In addition, although many research institutions adopted various tools to evaluate the NL of pregnant women in China, the lack of uniformity in these tools is not conducive to the comparison and integration of research results, which brought about the failure in providing well-grounded support for the formulation of relevant policies.

Therefore, our purpose is to develop the nutrition literacy assessment tool for Chinese pregnant women (NLAI-P), with the expectation of providing a unified tool for assessing and monitoring the staus of NL of pregnant women in China.

## 2. Materials and Methods

The study was carried out into 2 phases:

Phase1 was the establishment of NL core items for pregnant women in China.

**Figure 1:**
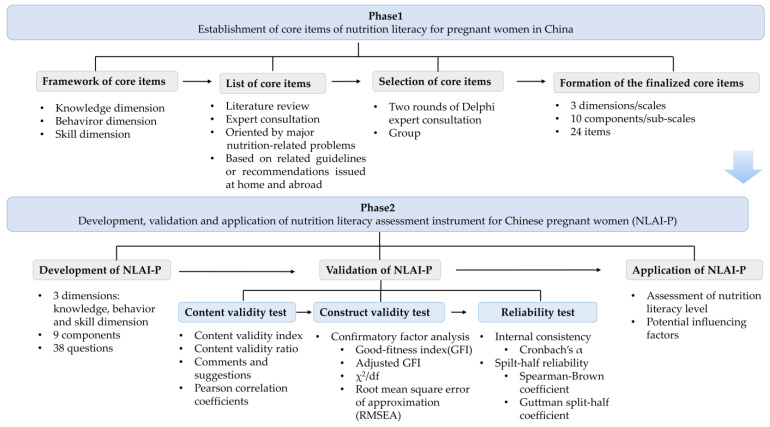
The flow chart of the study on the development, validation and application of nutrition literacy assessment for Chinese pregnant women (NLAI-P).

Phase2 was conducted for the development, validation and application of NLAI-P. The flow chart of the study was demonstrated in Figure 1.

Phase1: Establishment of NL core items for pregnant women in China.

The establishment of NL core items for Chinese pregnant women was divided into three steps. Firstly, the framework system of core items was determined. Based on the “Health Literacy of Chinese Citizens 66 Items-Basic Knowledge and Skills”, the framework system of NL core items for Chinese pregnant women was determined to contain 3 scales: basic knowledge and ideas, lifestyle and dietary behaviors, and basic skills. Secondly, a list of NL core items for Chinese pregnant women was built. Through literature review, expert consultation and group discussion, we identified item pool of NL for pregnant women by mainly referring to “Dietary Guidelines for Pregnant Women” issued by Chinese Nutrition Society along with recommendations and suggestions related to nutritional health of pregnant women issued by WHO. Thirdly, two rounds of Delphi expert consultation and group discussion were conducted to determine the final NL core items for Chinese pregnant women. The final version of NL core items for Chinese pregnant women consisted of 3 dimensions/scales (basic knowledge and ideas, lifestyle and dietary behaviors, and basic skills), 10 components/sub-scales (basic nutrition concept, food and nutrition knowledge, nutrition and disease knowledge, lifestyles, dietary behaviors, preparation for breastfeeding, gestational weight management, gestational disease management, acquisition, understanding and application of nutrition information, judgement of nutrition information, and nutrition decision making), and a total of 24 items.

The core items were scientific, reliable and has been previously published [19]. The NL core items for Chinese pregnant women were shown in Table 1.

Phase 2: Development, validation and application of NLAI-P.

### 2.1. The Development of NLAI-P

Based on above-mentioned NL core items for pregnant women, we developed NL questionnaire for Chinese pregnant women which consisted of 3 dimensions (knowledge, behavior and skill dimension), 9 components under dimensions and a total of 38 questions in components.

The NLAI-P consisted of two parts: the first part investigated demographical characteristics such as residence, age, career, education level, income and childbearing history. via a self-report questionnaire. The second part was NL questionnaire for Chinese pregnant women. The scoring standards of each question were detailed as follows:

The questions consisted of multiple choice and Likert type questions. The full score of a question was 2 points. For some multiple-choice questions with only one right responses, 2 points were awarded for the correct answer and 0 points for the wrong answer. For multiple choice questions with at least 2 correct responses, 2 points were given if respondents choose all correct answers, 1 point for people no choosing all right answers, and 0 for those picking up the wrong answer. For questions with five-point Likert scale (“strongly agree”, “agree”, “uncertain”, “disagree” and “strongly disagree”), If the question was positive, 2 points were rendered for “strongly agree”, 1 point for “agree” and 0 for “uncertain” or “disagree” or “strongly disagree”, vice versa. For questions with five-point Likert scale (“always”, “often”, “sometimes”, “occasionally” and “rarely”), if the question was positive, 2 points were awarded for “always”, 1 point for “often” and 0 for “sometimes” or “occasionally” or “rarely”, vice versa. The scores were categorized as excellent (>80% of total scores), good (60–80% of total scores) or poor (<60% of total scores).

### 2.2. The Validation of NLAI-P

#### 2.2.1. Data Collection

699 pregnant women were recruited by the convenience sample from 10 September 2020 to 31 December 2020 in certain hospitals in Beijing. The on-site investigation and online survey were carried out. Inclusion criteria were as follows: having signed the informed consent, being able to read and write, being apparently healthy with no severe diseases or mental disorders. Exclusion criteria were: being illiterate or having difficulty with verbal communication, withdrawing from the ongoing investigation. That the sample size was 5–10 times the number of questions was considered sufficient to perform the reliability and validity tests. The NLAI-P contained 38 questions, so the expected sample size was between 190 and 380. Therefore, the sample size in our study was acceptable.

The study was conducted according to the guidelines of the Declaration of Helsinki, and approved by the Committee on Medical Ethics of the Peking University with the number of ethics approval of IRB00001052-17107.

#### 2.2.2. Validity Test

(1) Content validity:

Questions were rated the relevance to the corresponding dimensions by using a five-point Likert scale (1 = completely unrelated, 2 = not relevant, 3 = some relevant, 4 = quiet relevant, 5 = highly relevant) in the expert consultation. We mailed experts the self-made “expert consultation questionnaire” and required each expert to appraise the relevance of each question. In addition, the experts were also asked to make comments and recommendations for optimizing the questions.

Content validity was assessed using the content validity index (CVI) and content validity ratio (CVR). CVI contained item-level CVI (I-CVI) for each question and scale-level CVI (S-CVI) for the scale. The I-CVI was calculated as the ratio of the number of experts that gave a relevance rating >3 to the total number of experts. The S-CVI was calculated as the average of I-CVI. I-CVI > 0.8 was considered the acceptability. The CVR was computed according to Lawshe scores which calculated CVR by subtracting half of the total number of experts from the number of experts rating the questions’ relevance more than 3, and the production dividing by the half of the total number of experts. According to the Lawshe scores table, CVR > 0.59 (for 11 experts) was considered acceptable [7,20].

Intra class correlation coefficient (ICC) was adopted to measure the consistency of experts’ ratings. ICC was computed by 2-way mixed effects ANOVA model by using IBM SPSS version 22.0 (SPSS, Inc., Chicago, IL, USA) and then divided as poor (<0.5), moderate (0.50–0.75), good (0.76–0.9) or excellent (>0.9).

Pearson correlation coefficient was computed to evaluate the correlation between the questions and the corresponding dimensions. The value of >0.4 was acceptable.

(2) Construct Validity test

The EFA was applied to test the construct validity of 3 dimensions. Kaiser Meyer Olkin measure of sampling adequacy (KMO) and Barrlett’s test of sphericity were performed to judge the suitability of EFA. KMO > 0.5 and *p* < 0.05 in Barrlett’s test of sphericity suggested the feasibility of EFA [21]. The number of factors was identified according to the Kaiser’s eigenvalue and scree plot. The questions with factor loadings > 0.25 were retained.

The CFA was used to test the fit degree of models derived from EFA. CFA is commonly implemented by structural equation modeling. The method of Weighted Least Squares was used for estimating the data. In CFA, the reasonable criteria of indices were as followings: goodness-of-fit indices (GFIs) > 0.9, adjusted goodness of fit index (AGFI) > 0.8, χ^2^/*df* < 3.0, and root mean square error of approximation (RMSEA) < 0.08 [11].

#### 2.2.3. Reliability

Internal reliability was commonly assessed through the Cronbach’α coefficient. It was derived from the formula:

$$\alpha =\frac{k}{k-1}\left(1-\frac{\sum S_{i}^{2}}{S_{T}^{2}}\right)$$

The *k* was the number of items. $S_{i}^{2}$ was the variance of the question *i*. The $S_{T}^{2}$ was the variance of scores of total items. Cronbach’*α* > 0.7 manifested the good acceptability [22].

The spilt-half reliability was another indicator used for evaluating the reliability [23]. The questionnaire was randomly divided into two parts, and then Spearman-Brown and Guttman split-half coefficients were calculated between the scores of two parts.

### 2.3. The Application of NLAI-P

To evaluate the NL level of pregnant women in China and analyze the potential influencing factors.

### 2.4. Statistical Analysis

Data processing and calculating was performed by using software SPSS25.0 (SPSS Inc., Chicago, IL, USA). One-way analysis of variance (ANOVA) or T test was used to compare the differences in NL scores in different groups. Multiple linear regression model was established for determining the factors related to NL level of Chinese pregnant women. The CFA was carried out by using software AMOS25.0 (SPSS Inc., Chicago, IL, USA).

## 3. Results

### 3.1. The Dimension and Components of NLAI-P

The framework of NLAI-P was finally defined with 3 dimensions: knowledge dimension, behavior dimension and skill dimension. Each dimension consisted of 5, 2 and 3 components, respectively (shown in Table 2).

### 3.2. Demographic Characteristics in Confirmatory Study

699 pregnant women were recruited. The demographic characteristics were shown in Table 3. Their average age was 31.2 years old. 94.1% of pregnant women were ethnic Han. There were 87.8%, 9.9% and 2.4% of pregnant women from eastern, middle and western regions of China, respectively. 48.7% of them had the education level of master degree or above. The proportion of pregnant women were 25.2%, 36.3% and 38.8% in early, middle and late trimester, respectively. 49.2% of pregnant women was first-time mothers.

### 3.3. Content Validity

#### 3.3.1. I-CVI and CVR

The Table 4 presented that the I-CVI of NLAI-P ranged from 0.71 to 1.0 with the S-CVI of 0.97. In knowledge dimension, the I-CVI was from 0.85 to 1.0 denoting the acceptable content validity, while the I-CVI of 0.7–1.0 in behavior dimension and skill dimension connoted barely satisfactory content validity. ICC suggested the moderate consistency of experts’ ratings.

#### 3.3.2. Pearson Correlation Coefficient

The result of Pearson correlation coefficients > 0.4 implied the acceptable content validity of NLAI-P (shown in Table 5).

### 3.4. Construct Validity Test

#### 3.4.1. Exploratory Factor Analysis

In NLAI-P, the KMO in 3 dimensions were 0.76, 0.74 and 0.77, respectively, and Bartlett’s test of sphericity of *p* < 0.001, which implied the feasibility of EFA. In knowledge dimension, 4 factors were extracted and accounted for 32.12% of total variance. They represented food, nutrition and health, risk factors of pregnancy complication, knowledge of balanced diet and healthy lifestyles, and weight management. questions loaded between 0.22–0.77 in the dimension. In behavior dimension, 2 factors were obtained, accounting for 40.95% of the total variance, and they were named as healthy eating behaviors and healthy lifestyles. All of the questions had factor loadings >0.25. In skill dimension, 3 factors were identified, accounting for 60.70% of the total variance, and were named after food group and nutrition label analysis, judgement of nutrition information recognition, nutrition information access and nutrition-related decisions making, respectively. Factor loadings of each question kept up to the acceptable standard.

#### 3.4.2. Confirmatory Factor Analysis

CFA was used to evaluate the structure fit degree of NLAI-P, and the results were shown in Table 6. The GFI of the overall NLAI-P was 0.86, close to the nominal value of 0.9, and RMSEA was excellent with a value of 0.046. Each index of 3 dimensions dropped into the acceptable range, which meant that the fitness of the 3 dimensions was very good.

### 3.5. Reliability and Validity

#### Internal Consistency and Spilt-Half Reliability

Cronbach’s α coefficient of the overall evaluation tool was 0.82, and Spearman-Brown and Guttman split-half coefficients were 0.73, which could be interpreted as a good internal reliability of the NLAI. Cronbach’s α coefficient were 0.72, 0.60 and 0.68 in knowledge, behavior and skill dimension, respectively (shown in Table 7).

### 3.6. Assessing Nutrition Literacy for Pregnant Women in China and Its Related Factors

#### 3.6.1. The NLAI-P Scores of Chinese Pregnant Women

The average NLAI-P scores were 46.59 ± 9.27. Only 3.9% of pregnant women reached the excellent level, more than half (55.2%) of pregnant women had good nutritional literacy level, and 40.9% of pregnant women were in poor level (shown in Table 8). As for the scores of each dimension, the excellence rate of knowledge dimension and behavior dimension is less than 5%, the excellent rate of skill dimension is 47.1%, and 77.8% of pregnant women were at poor level in behavior dimension (shown in Table 8).

#### 3.6.2. The Potential Factors Influencing Nutrition Literacy of Chinese Pregnant Women

The potential factors influencing the NL level of pregnant women included age, ethnicity, residence, occupation, education level and gestational age (shown in Table 9). Some demographical characteristics affected the level of NL of pregnant women except for ethnic group and gestational age. Age, residence, occupation, and education level as well as parity affected the overall scores of NLAI-P and knowledge dimension. The scores of behavior dimension were related to age, occupation and education level. The factors including residence, occupation, and education level had impacts on the scores of skill dimension. Pregnant women less than 25 years old had the lowest scores, which were lower than those in older groups. The higher scores occurred among pregnant women from eastern regions rather than those living the middle regions. With the advancement of education level, the scores increased. Comparing to those being pregnant more than one time, first-time mothers obtained higher scores of nutrition literacy.

**Figure 2:**
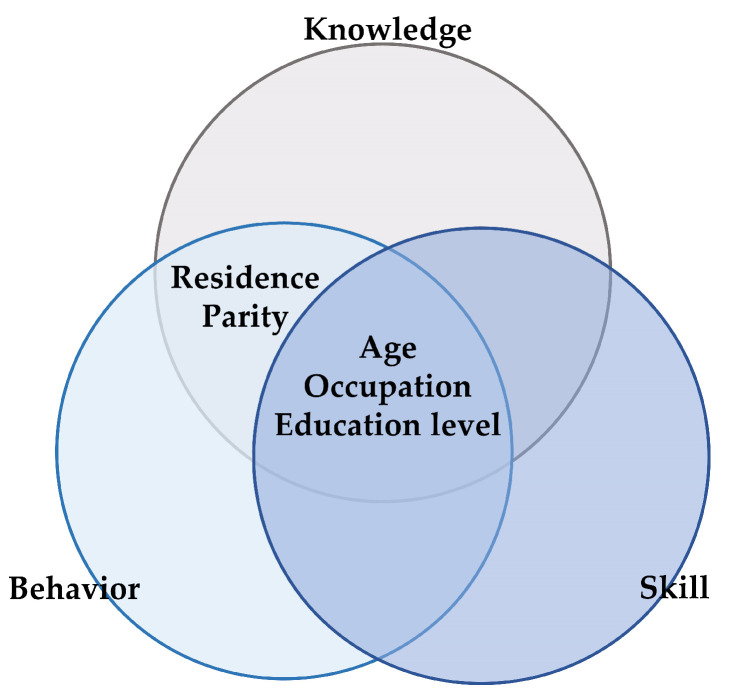
Venn diagram of influencing factors of nutritional literacy scores in Chinese pregnant women.

The Veen diagram showed that age, occupation and education level had impacts on all of 3 dimensions (shown in Figure 2).

Then, the multiple linear regression model was established by using the potential factors including age, residence, occupation and education level. The results showed that the education level presented significantly positive correlation with both the overall scores of NLAI-P and the scores of all dimensions (shown in Table 10). As the level of education increased, the scores rose up in the NLAI-P (β = 3.67, *p* < 0.01), knowledge dimension (β = 1.98, *p* < 0.01), behavior dimension (β = 0.69, *p* < 0.01) and skill dimension (β = 0.99, *p* < 0.01).

## 4. Discussion

The NLAI-P in the study consisted of 3 dimensions, 9 components and a total of 38 questions, and possessed the satisfactory validity and reliability. The NLAI-P appeared as a valid and reliable tool to assess NL level for Chinese pregnant women.

The overall content validity of NLAI-P was 0.98, which suggested that the instrument was well designed to measure NL of Chinese pregnant women. The results of CFA also implied that the acceptable fit indices for the proposed models of NLAI-P. Our NLAI-P contained 3 dimensions: knowledge dimension, behavior dimension and skill dimension. Under knowledge dimension, there were 4 components: food and nutrition knowledge, nutrition and health knowledge, balanced diet and healthy lifestyles, and weight management as well as risk factors of pregnancy complications. The dimension was designed for evaluating whether pregnant women learnt balanced diet, whether to master the knowledge about the optimal food source of maternal essential nutrients (iron, iodine and folic acid) and about the adverse pregnancy outcomes induced by these nutrients’ deficiency, and whether to know the detrimental aspects of excessive/insufficient gestational weight gain as well as identifying the risk factors of pregnancy complications. Similarly, food, nutrition and health also appeared in the existing nutrition literacy assessment tools for school children [7,18], the old population [14,17] and the general adults [14,16]. Gibbs stated that there was great necessity to popularize knowledge related to the considerable effects of nutrient intake on diseases and health during the course of nutrition education [1,13,24]. Diamond developed NLS for general adults, and also included the heart disease related diet into the scale [12]. The Food and Nutrition Literacy Questionnaire for Chinese Adults (FNLQ) developed by Zhang et al. also encapsulated the food and nutrition knowledge [16]. The components of balanced diet and healthy lifestyle knowledge was designed to test whether to grasp the knowledge of rational diet, sufficient water drinking and proper physical activities during pregnancy. The component of weight management focused on monitoring gestational weight because of the crucial role that appropriate gestational weight gain played in optimal pregnancy outcomes. The component of risk factors related to pregnancy complications underlined the high hazard factors contributing to gestational diabetes mellitus and hypertension syndrome in pregnancy, both of which were prevalent among pregnant women in China [9]. Identification and avoidance of these risk factors was conducive to the primary prevention of diseases. The I-CVI > 0.8 illustrated the desirable content validity in knowledge dimension, which implied that the items corresponded to what the dimension was designed to test.

For behavior dimension with 2 components, one was healthy eating behaviors and the other was healthy lifestyles. The former aimed at inspecting whether respondents followed healthy eating behaviors such as how many kinds of food intake, the consumption of milk and dairy products, water drinking, et al. The component of healthy lifestyles was designed for evaluating whether pregnant women adopted healthy lifestyles such as taking proper exercise, paying attention on gestational weight gain and interpreting nutrition label. In accordance with ours, items concerning nutrition label also appeared in the existing nutrition literacy tools [2,7,17,18,25]. The dimension also had good content validity.

For the skill dimension, it included 3 components representing food group and nutrition label analysis, the judgement of nutrition information and nutrition information access and nutrition-related decision making. For the component of food group and nutrition label analysis, classifying food correctly was the prerequisite and foundation for choosing a healthy meal, which was in line with previous assessment tools covering the skill of food classification [2]. Nutrition label interpretation was devised for evaluating the numeracy and reading ability. Since the ability played a critical role in understanding the various nutrient content in food, and then instructing consumers to make the optimum food choice, most of the existing nutrition literacy scales encapsulated relevant items for assessing the capability of the food labels/nutrition label analysis and comprehension [26,27,28]. The judgement of nutrition information was the embodiment of the interactive nutrition literacy and to test whether pregnant women was capable of identifying and judging various sources of nutrition information and knowledge in daily social activities [29,30]. Just as excellent health literacy, advanced nutrition literacy does not only mean being equipped with the ability of reading, writing and understanding as well as applying the nutrition information, but also being able to filter, judge and evaluate nutrition information [4]. Nutrition-related decisions making was defined as the ability of pregnant women to transform nutrition knowledge and information into actions so as to address their own nutrition-related issues or barriers, which belonged to the highest level of nutrition literacy, namely the category of the critical nutrition literacy according to Velardo S [3].

Our NLAI-P had a satisfactory Cronbach’s α of 0.82 indicating the acceptable internal consistency. NL assessment tools designed for Chinese adults, elderly and school-age children had acceptable Cronbach’s of 0.89, 0.68 and 0.69, respectively [16,17,18]. NLS developed by Diamond for American adults had the Cronbach’s α of 0.83 and contained 28 items covering diet related to heart health, saturated fatty acids, food portion, organic food, dietary fiber, calcium and sugar [12]. FNLIT was developed for Iran elementary school children and was divided into 2 domains (cognitive domain and skill domain) involved in 6 facets (understanding and knowledge; functional, food choice, interactive, and critical skills) and 46 items in total with the Cronbach’s α over 0.7 [7]. Chau et al. established the health literacy scale for low salt consumption to evaluate the salt intake on the basis of 3 domains of health literacy. The scale contained 8 facets and in total of 49 items with the good reliability (Cronbach’s α = 0.8) [31].

The results indicated that the average scores of NLAI-P was 46.59. Currently, there was no unified standards to evaluate whether to have sufficient NL because of different NL assessment tools for different target population. In this study, the standards were set by referring to health literacy evaluation standard officially issued in China [32]. The scores of <= 80% of total scores were considered as excellence, 60–80% rated as good, while scores below 60% regarded as poor. The results showed that only 3.9% of pregnant women reached the excellent level, indicating that inadequate NL was common among Chinese pregnant women, and the improvement of NL of pregnant women should not be ignored. Other studies using self-made questionnaires have also found that Chinese pregnant women had insufficient nutrition knowledge [33,34], although these questionnaires tended to only survey nutritional knowledge rather than comprehensive NL assessment tools. In knowledge dimension, 60% of pregnant women scored 60–80% of the total scores of dimensions, indicating that most pregnant women had a slight deficiency in nutrition knowledge, while in behavior dimension, nearly 78% (77.8%) of pregnant women scored below 60% of the total scores. That implied that the gap existed between knowledge and behavior. Additionally, it also indicated that simple detection of pregnant women’s nutrition-related knowledge level was not enough, and the investigation and monitoring of pregnant women’s nutrition-related behavior should also arouse considerable attention.

In the current study, age, occupation and education level came as the main factors affecting NL of pregnant women. Compared with those under 25 years old, the pregnant women in the older group had higher scores whether in overall NLAI-P or 3 dimensions, which might be related to young people’s lack of focus on nutrition-related knowledge, behavior and skills. Occupation was another influencing factor, and the scores of professional and technical personnel were the highest. It was considered that this population may have more access to NL related sources, and on the other hand, they tended to possess higher education level, which was supported by the positive correlation between education level and NLAI-P scores in our study. After adjusting the confounding factors, there still existed significantly positive association between education level and NLAI-P scores. In consistent with our findings, Portuguese adults with higher education were reported having higher levels of NL [35]. Likewise, Zhang et al. also founded that well-educated people performed better in NL survey [16]. The findings informed us that the propaganda and education on nutrition and health should be tailored to the education level of pregnant women, in order to achieve optimized intervention effects.

There are some advantages in our study. Firstly, it was the first instrument to evaluate the NL of Chinese pregnant women. We conducted the reliability and validity tests in a sufficiently large sample size and acquired desirable results. Secondly, we adopted the well-established theory as the basis of theoretical framework. At the same time, we extensively referred to the dietary guidelines issued by Chinese Nutrition Society and other reports, recommendations as well as guidelines published by WHO or other official organizations as the content basis, and were oriented by the main nutritional and health problems of Chinese pregnant women. Therefore, there were good grounds for us to state that NLAI-P was supported by the solid empirical and theoretical basis.

Undoubtedly, there were some limitations we acknowledged. The application of NLAI-P was restricted by the certain social and cultural environment, and whether it can be popularized in the world remains uncertain. Furthermore, there is still some room left for conducting research on the association between NL with pregnancy outcomes.

## 5. Conclusions

NLAI-P was valid and reliable for evaluating the NL level of Chinese pregnant women. The development of NLAI-P would provide an effective tool for assessing and monitoring the status of nutrition literacy among pregnant women and render a basis on the designation of targeted nutrition interventions.

## Figures and Tables

**Table 1:** The core items of nutrition literacy for pregnant women in China.

Scales	Sub-Scales	Items
Basic knowledge and ideas	Basic nutrition concept	1. Reasonable nutrition during pregnancy has crucial impacts on the short-term and long-term health of both mothers and children.
		2. Appropriate gestational weight gain contributes to optimal pregnancy outcomes.
		3. Smoking and drinking during pregnancy are likely to cause miscarriage, premature and fetal malformation.
	Food and nutrition knowledge	4. Milk is rich in calcium and easily absorbed, making it an ideal food source of calcium.
		5. Animal liver, eggs, legumes, green leafy vegetables, fruits and nuts are good food sources of folic acid.
	Nutrition and disease knowledge	6. Insufficient dietary iron intake very likely leads to iron deficiency or iron deficiency anemia in pregnant women and infants.
		7. Iodine deficiency during pregnancy can impair the brain and intellectual development of the fetus.
		8. Increased intake of food rich in dietary fiber during pregnancy can help relieve constipation in pregnant women.
		9. Insufficient carbohydrate intake in early pregnancy can impair the development of fetal nervous system.
Lifestyle and dietary behaviors	Lifestyles	10. At least 30 min of physical activity should be carried out every day during pregnancy if physically able, and vigorous exercise and heavy labor should be avoided.
	Dietary behaviors	11. Keep foods diversified and nutrition balanced during pregnancy.
		12. From 3 months before pregnancy, 400 μg folic acid supplements used can prevent fetal neural tube development deformity.
		13. In severe cases of morning sickness, it is not necessary to overemphasize balanced diet, but to ensure adequate intake of cereals and tubers.
		14. Ensure adequate daily intake of water intake and avoid or limit beverages containing sugar, caffeine during pregnancy.
		15. Eat deep-sea fish 2–3 times per week during pregnancy to provide the fetus with n-3 long-chain polyunsaturated fatty acids that play an important role in retinal development.
		16. Increase properly the intake of iron-rich animal food in the second and third trimesters and eat animal blood and liver 1–2 times per week.
		17. Choose iodized salt, often eat iodine-rich seafood such as kelp, laver.
		18. From the second trimester, increase intake of milk by 200 mL per day in order to make total intake of milk reach 400–500 mL per day.
	Preparation for breastfeeding	19. Pregnant women should actively prepare for breastfeeding and learn the methods and skills of successful breastfeeding.
Basic skills	Gestational weight management	20. Monitoring and managing your weight before pregnancy. Measure gestational weight once a month in early pregnancy and once a week in the second and third trimester.
	Gestational disease management	21. Pay attention to blood glucose changes and judge the risk factors of diabetes. Pregnant women with gestational diabetes mellitus should strengthen the skill in disease self-management.
		22. Pay attention to blood pressure changes and judge the risk factors of hypertension. Pregnant women with hypertension syndromes should strengthen the skill in disease self-management.
	Judgement of nutrition information, and nutrition decision making	23. Read and understand food labels and choose packaged food wisely.
	Acquisition, understanding and application of nutrition information	24. Pay attention to nutrition-related information during pregnancy, and be able to obtain, understand, screen and apply nutrition information during pregnancy.

**Table 2:** The dimension and components of NLAI-P.

Dimension	Components	Questions
Knowledge	food, nutrition and health	11
	knowledge of balanced diet and healthy lifestyles	5
	weight management	5
	risk factors of pregnancy complications	2
Behavior	healthy eating behaviors	3
	healthy lifestyles	4
Skill	food group and nutrition label analysis	4
	judgement of nutrition information	2
	nutrition information access and nutrition-related decisions making	2
NLAI-P		38

**Table 3:** Demographic characteristics of respondents ^a^.

Variables	*n* = 699
Age	31.2 ± 4.1
<25	28 (4)
25–30	215 (30.8)
30–35	346 (49.5)
>35	104 (14.9)
Height (cm)	162.6 ± 5.1
Pre-pregnancy body weight (g)	117.6 ± 20.7
Body weight (g)	130.7 ± 23.6
Pre-pregnancy BMI (kg/m^2^)	22.2 ± 3.6
BMI (kg/m^2^)	24.7 ± 4.2
Gestational weight gain (g)	6.5 ± 5.8
Ethnicity	
Han	658 (94.1)
Non-Han	38 (5.4)
Residence	
East	613 (87.7)
Middle	69 (9.9)
West	17 (2.4)
Career	
Housewife	151 (21.6)
Civil servant	108 (15.5)
Professional	179 (25.6)
Service industry	121 (17.3)
Others	140 (20)
Education	
Junior high school or below	21 (3)
Senior high school	74 (10.6)
University	263 (37.6)
Master degree or above	341 (48.7)
Parity	
1	344 (49.2)
≥1	355 (50.8)
Gestational week	
1st TM ^b^	176 (25.2)
2nd TM	254 (36.3)
3rd TM	269 (38.5)

^a^ Mean (standard deviation) for the continuous variables, N (%) for the categorized variables. ^b^ TM: trimester.

**Table 4:** The CVI and CVR of each dimension and the whole NLAI-P.

Dimension	S-CVI	CVR	I-CVI	ICC (95% CI)
Knowledge	0.99	0.98	0.85–1.0	0.54 (0.28, 0.73)
Behavior	0.98	0.96	0.71–1.0	0.63 (0.24, 0.87)
Skill	0.97	0.95	0.71–1.0	0.74 (0.42, 0.92)
NLAI-P	0.98	0.97	0.71–1.0	0.61 (0.43, 0.76)

**Table 5:** Pearson correlation coefficients of NLAI-P.

Dimension	Knowledge	Behavior	Skill
Knowledge			
Behavior	0.43		
Skill	0.54	0.44	
NLAI-P	0.92	0.67	0.78

**Table 6:** Confirmatory factor analysis of NLAI-P.

Dimension	χ^2^/*df*	GFI	AGFI	RMSEA
NLAI-P	1.82	0.86	0.84	0.046
Knowledge	1.61	0.93	0.92	0.039
Behavior	1.09	0.99	0.98	0.012
Skill	1.49	0.98	0.97	0.035

**Table 7:** Cronbach’s α coefficient and spilt-half reliability of NLAI-P.

Dimension	Cronbach’s α Coefficient	Spilt-Half Reliability	
		Spearman Brown	Guttman Split-Half
Knowledge	0.72	0.68	0.68
Behavior	0.65	0.67	0.67
Skill	0.68	0.59	0.52
NLAI-P	0.82	0.73	0.73

**Table 8:** The NLAI-P scores of pregnant women in China.

Dimensions	Number of Questions	Total Scores	Scores	Minimum	Maximum	≥80% *n* (%)	60–80% *n* (%)	<60% *n* (%)
Knowledge	23	46	28.6 ± 5.67	2.00	42.00	33 (4.7)	424 (60.7)	242 (34.6)
Behavior	7	14	6.4 ± 2.69	0.50	16.00	30 (4.3)	125 (17.9)	544 (77.8)
Skill	8	16	11.59 ± 3.02	1.90	16.00	329 (47.1)	212 (30.3)	158 (22.6)
NLAI-P	38	76	46.59 ± 9.27	12.00	68.00	27 (3.9)	386 (55.2)	286 (40.9)

**Table 9:** The potential factors influencing NL of Chinese pregnant women.

Variables	NLAI-P		Knowledge		Behavior		Skill	
	Scores	*p*	Scores	*p*	Scores	*p*	Scores	*p*
Age		0.003		0.004		0.019		0.126
<25 (reference)	40.3 ± 10.5		25 ± 5.9		4.9 ± 2.6		10.3 ± 3.3	
25–30	46.9 ± 8.7 *		28.8 ± 5.3 *		6.3 ± 2.8 *		11.8 ± 2.8 *	
30–35	47 ± 9.2 *		28.9 ± 5.7 *		6.5 ± 2.6 *		11.6 ± 3 *	
>35	46.1 ± 9.6 *		28.1 ± 5.9 *		6.6 ± 2.6 *		11.5 ± 3.1	
Ethnicity		0.554		0.360		0.607		0.167
Han (reference)	46.4 ± 9.3		28.5 ± 5.7		6.4 ± 2.7		11.5 ± 3	
Non-Han	48.8 ± 8.2		29.7 ± 5.1		7 ± 2.9		12.2 ± 2.5	
Residence		0.016		0.045		0.410		0.010
East (reference)	46.9 ± 8.9		28.8 ± 5.6		6.5 ± 2.6		11.7 ± 2.9	
Middle	43.6 ± 11.3 *		27 ± 6.6 *		6 ± 3.1		10.6 ± 3.4 *	
West	46.7 ± 10.2		29.1 ± 4.9		6.3 ± 3		11.3 ± 3.8	
Career		0.000		0.000		0.003		0.000
Housewife (reference)	43 ± 9.7		26.5 ± 6.2		5.8 ± 2.5		10.7 ± 3.3	
Civil servant	47.6 ± 9.5 *		29 ± 5.7 *		6.8 ± 3 *		11.9 ± 3.1 *	
Professional	49.7 ± 8.5 *		30.5 ± 5.1 *		6.9 ± 2.7 *		12.3 ± 2.5 *	
Service industry	45.2 ± 9.4 *		27.6 ± 5.4		6.3 ± 2.9		11.2 ± 3.3	
Others	46.9 ± 7.9 *		29 ± 5.1 *		6.2 ± 2.4		11.6 ± 2.8 *	
Education		0.000		0.000		0.000		0.000
Junior high school or below (reference)	39.1 ± 10.4		24.4 ± 7		4.8 ± 2.3		9.8 ± 3	
Senior high school	40.3 ± 10.7		25.3 ± 6.2		5.4 ± 2.5		9.7 ± 3.7	
University	46 ± 8.8 *		28.3 ± 5.5		6.3 ± 2.7		11.5 ± 3	
Graduate or up	48.9 ± 8.3 *		29.8 ± 5.2 *		6.8 ± 2.6 *		12.2 ± 2.6 *	
Parity		0.012		0.011		0.618		0.013
1 (reference)	47.5 ± 8.8		29.2 ± 5.4		6.5 ± 2.7		11.9 ± 2.9	
≥1	45.7 ± 9.6		28.1 ± 5.9		6.4 ± 2.6		11.3 ± 3.1	
Gestational week		0.900		0.307		0.070		0.648
1st TM(reference)	46.5 ± 9.7		28 ± 6.1		6.7 ± 2.7		11.8 ± 3.1	
2nd TM	46.4 ± 9.6		28.8 ± 5.8		6.1 ± 2.7		11.5 ± 3	
3rd TM	46.8 ± 8.7		28.8 ± 5.2		6.5 ± 2.7		11.5 ± 2.9	

* comparing with the reference, *p* < 0.05.

**Table 10:** The correlation coefficients between education level and the NLAI-P scores of Chinese pregnant women.

Variable	NLAI-P		Knowledge		Behavior		Skill	
	*β*	*p*	*β*	*p*	*β*	*p*	*β*	*p*
Education level	3.67 *	0.000	1.98 *	0.000	0.69 *	0.000	0.99 *	0.000

* *p* < 0.05.

## Data Availability

The datasets used during the current study are available from the corresponding author on reasonable request.
